# Recall cues interfere with retrieval from visuospatial working memory

**DOI:** 10.1111/bjop.12374

**Published:** 2019-01-02

**Authors:** Younes Adam Tabi, Masud Husain, Sanjay G. Manohar

**Affiliations:** ^1^ Nuffield Department of Clinical Neurosciences University of Oxford UK; ^2^ Department of Experimental Psychology University of Oxford UK

**Keywords:** visuospatial working memory, retrieval, recall cues

## Abstract

Visuospatial working memory allows us to hold multiple visual objects over short delays. It is typically tested by presenting an array of objects, then after a delay showing a ‘probe’ indicating which memory item to recall or reproduce by adjusting a target feature. However, recent studies demonstrate that information at the time of probe can disrupt recall. Here, in three experiments we test whether traditional memory probes, which contain features that compete with the feature to be recalled, may themselves interfere with performance. We asked participants to report the direction of one of the several coloured arrows in memory, based on its colour. First, we demonstrate that recall is better when the probe is initially just a coloured dot, rather than a coloured arrow which has to be adjusted to match orientation memory, consistent with interference from features of the probe itself. Second, this interference is present even when a mask follows the memory array, suggesting that the interference does not work by degrading immediate or iconic memory. Finally, when items are shown sequentially, the first and last items are invulnerable to probe interference. Our findings support recent theories of associative recall, in which probes reactivate features in WM, retrieving information by pattern completion.

## Background

Interference is considered to be a crucial cause of forgetting in working memory (WM; Oberauer & Lin, 2017). For example, recall errors can be biased towards reporting the features of other items held in memory, an effect that increases with the number of items that are encoded into WM and duration of maintenance (Pertzov, Manohar, & Husain, 2017). A further intriguing possibility is that interference could arise at the time of the retrieval due to the probe used to cue recall.

In many WM studies, the probe which is used at retrieval often contains attributes that might interfere with the feature that has to be recalled. For example, in adjustment paradigms in which participants are asked to report the direction of an arrow, the probe is usually an arrow the direction of which must be subsequently adjusted to match the memory of its orientation (Gorgoraptis, Catalao, Bays, & Husain, 2011; Pertzov *et al*., 2017; Zokaei, Heider, & Husain, 2014). In principle, this arrow could itself interfere with stored items, since it holds information of a similar type. The probe may therefore introduce additional information over and above the stored memory array that could interfere with memory recall.

One demonstration that information presented at test interferes with memory retrieval comes from the retro‐cue effect (see Souza & Oberauer, 2016, for a review). In the retro‐cue paradigm, a cue (usually an arrow) is shown during the retention interval indicating the location of the item that will be tested at the end of the trial. The retro‐cue provides no information about the memorandum's feature or about the response, thereby only allowing participants to retrieve information from memory using the location associated with the item. Performance in retro‐cue trials is better than performance in trials in which memory is tested directly (i.e., no‐cue trials).

One explanation of this effect is that retro‐cues allow participants to retrieve information in the absence of the interference inflicted by the memory probe. To provide evidence for this hypothesis, Souza, Rerko, and Oberauer (2016) included a condition in which the probe was shown at the same time as the retro‐cue, but participants had to delay making a decision to the probe. In this new probe + delay condition, the probe could interfere with memory retrieval, but participants were still forced to invest more time in retrieving information from memory as in the retro‐cue condition. Although the probe + delay condition also improved accuracy compared to the no‐cue condition, this effect was smaller than in the retro‐cue condition, indicating that one component of the retro‐cue benefit is protection from probe interference. Protection from interference was also demonstrated in a task in which participants had to reproduce the memorandum's feature (i.e., colour) in a continuous scale by using a colour wheel. Souza *et al*. (2016) observed that part of the erroneous responses in this task were due to an increased probability of selecting colours in the colour wheel that appeared next to the probed location (a colour wheel attraction effect). This attraction effect was extinguished in the retro‐cue condition, which delayed the presentation of the colour wheel until retrieval of the probed item was already substantially advanced. In line with the idea that the colour wheel interferes with memory retrieval, replacing the coloured wheel by a grey wheel led to an overall improvement in performance even in the absence of a retro‐cue, and likewise, removing this source of interference also reduced the retro‐cue benefit.

**Figure 1:**
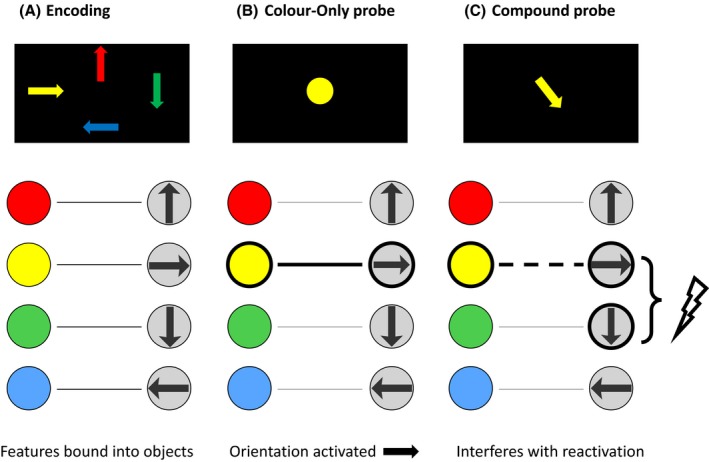
Schematic of pattern completion with a colour‐only versus a compound probe. (A) When participants are presented with a memory array and are asked to keep the items’ colours and orientations in mind, they encode both dimensions for each item and bind them together into objects. After a memory delay, participants are presented with a either colour‐only probe that only holds information on one dimension or compound probe that holds information on two dimensions. (B) In case of the colour‐only probe, this colour cue can reactivate the corresponding orientation information, by pattern completion. This could allow participants to report the appropriate item's orientation (Manohar *et al*., 2017). The bold line represents activation though pattern completion. (C) However, when participants are presented with a compound probe, such as used in many previous studies, the probe holds not only the colour cue, but also irrelevant information on an orientation that is different from the encoded item. This additional information could interfere with reactivating items from memory (dashed bold line). In this example, the probe's orientation might interfere with irrelevant information previously stored such as another orientation (flash). [Colour figure can be viewed at wileyonlinelibrary.com]

Can a common mechanism explain why probes interfere with recall, but also why retro‐cues confer an advantage even if they result in increased delays? An associative pattern completion mechanism (Figure 1) would predict that the relevant feature of the probe triggers reactivation of the other features belonging to the appropriate item in memory, allowing them to be recalled (Manohar, Zokaei, Fallon, Vogels, & Husain, 2017). Crucially, this process of pattern completion would be expected to be disrupted if the probe also activated a competing feature on the dimension to be recalled that was not actually in the object being recalled. In this way, the presence of an additional irrelevant perceptual feature might act as an obstacle to retrieval. Thus, the additional feature is predicted to interfere with associative retrieval.

If such probes do indeed interfere with retrieval, then many tasks which aim to measure memory capacity, such as the adjustment/reproduction paradigms, might actually systematically underestimate memory capacity (Bays & Husain, 2008; Zhang & Luck, 2008). Moreover, interference arises in many cognitive domains including response selection, planning, and dual‐task processing (Schumacher *et al*., 2018). These cognitive functions are also underpinned by WM (Shallice & Burgess, 1996), and unimpeded recall of task‐relevant information from WM may be a crucial component of executive function in general. Thus, interference effects observed at the time of WM recall could potentially shed light on interference effects in many other domains. In particular, interference at the time of a memory probe could also potentially explain interference in situations involving task rules. Storing and executing task rules may be homologous to WM storage and recall, respectively, and may involve similar pattern‐completion mechanisms.

In the current study, we asked whether reducing the interaction between the probe and memory items – by removing the irrelevant feature from the probe – improves performance. We employed a new reporting method in which the probe contains *only the relevant cue feature* that indicates which memory item to recall – rather than combining the cue feature with an irrelevant feature – while still providing an analogue measure of memory precision. For example, if the task is to recall the direction of a coloured arrow, we initially show a central coloured dot as a probe, to indicate which item must be recalled, rather than a coloured orientated arrow that must be adjusted. To report the remembered arrow's direction, participants moved the mouse in the desired direction. Once the mouse moved, an arrow appeared pointing so that the direction could subsequently be adjusted. We hypothesized that when their memory was interrogated with such a ‘bare’ (dot) probe, participants would be able to give more precise responses compared to the usual situation in which a coloured arrow is presented right from the start of the probe period.

One possible explanation for any interference identified at the time of probe could be that the probe arrow somehow ‘overwrites’ perceptual information stored in a shorter term visual buffer such as iconic memory. If there were such iconic memory buffer (Kosslyn, 1980; Sperling, 1960) that supplements WM precision, the visual arrow probe might interfere with that perceptual store. Thus, in a second experiment we tried to erase any such buffer by a visual mask. So, if probe interference operates by disrupting iconic memory, then masking would be expected to reduce the degree to which the probe degrades performance.

Finally, according to the pattern‐completion hypothesis, probe interference should arise when items must be reactivated from memory (Manohar *et al*., 2017). But several studies have demonstrated that one item in memory may be held in a privileged state, which may be more active than other stored items (Lewis‐Peacock, Drysdale, Oberauer, & Postle, 2012; Stokes *et al*., 2013; Wolff, Jochim, Akyürek, & Stokes, 2017; Zokaei, Manohar, Husain, & Feredoes, 2014; Zokaei, Ning, Manohar, Feredoes, & Husain, 2014). For example, the last item in a sequence is recalled more easily – the ‘recency effect’ – perhaps because its memory remains in the active state (Gorgoraptis *et al*., 2011; Nee & Jonides, 2008, 2011, 2013; Oztekin, Davachi, & McElree, 2010; Zokaei, Gorgoraptis, Bahrami, Bays, & Husain, 2011). If reactivation were only required for earlier items in a sequence, a clear prediction is that interference from the probe should only affect the earlier items.

## Materials and Methods

### Participants

We tested 15 participants in Experiment 1 (four male; *M* = 25.47; *SD* = 3.68; one left‐handed); 15 individuals (10 male; *M* = 26.73 years; *SD* = 4.37; 0 left‐handed) in Experiment 2; and another 15 participants (eight male; *M* = 25.8 years; *SD* = 3.28; one left‐handed) in Experiment 3. All were neurologically normal and naive to the purpose of the experiment. The study was approved by the local ethics committee, and participants gave their informed consent. Analysis scripts and data can be found on OSF (https://osf.io/3fqsu/?view_only=791a3b57ac444340b15b34293119e403).

### Experiment 1: Simultaneous presentation

#### Stimuli

Stimuli were presented at a viewing distance of approximately 60 cm on a 24‐inch LCD monitor. Each memory array consisted of differently coloured arrows (approximately 2.5° × 0.4° of visual angle) that were chosen from eight easily distinguishable colours (red, green, blue, yellow, cyan, violet, white, and orange) presented on a black background, spaced along the boundary of an imaginary circle (radius 6.5°) around fixation with equal interitem distances (centre to centre). All arrows within a single memory array were differently coloured and were oriented in random directions (angles distributed uniformly over 360°). Specifically, randomization was achieved using MATLAB ‘rand’ function to sample values continuously and uniformly between 0 and 1 and then multiplying those by 2*π.

#### Procedure

**Figure 2:**
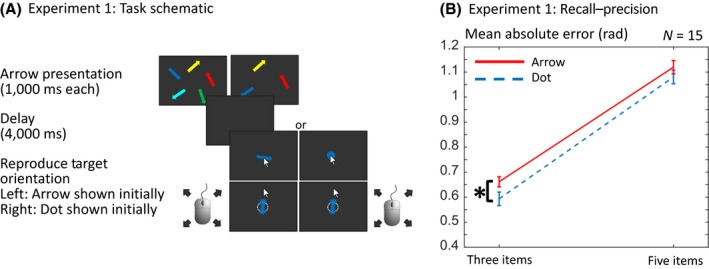
Design and mean absolute error for Experiment 1. (A) Participants had to remember the orientations of a set of coloured arrows. Three or five items were presented in the memory array, and after a delay, one of the arrows was probed by their colour. In the ‘arrow probe’ condition, the probe was a compound object, that is, a coloured arrow that had to be adjusted to the remembered orientation, by moving the mouse. In the ‘dot probe’ condition, the probe was a coloured dot (no irrelevant orientation information, colour only), which turned into an arrow as soon as the mouse was moved. (B) The arrow probe significantly impaired recall–precision (*p* = .029). As expected, memory was worse when more items had to be stored (*p* < .001), but there was a significant interaction between the probe type and number of items in the array. The error bars were calculated by subtracting each subject's grand mean away from their individual per‐condition values and showing ± the standard error (Loftus & Masson, 1994). [Colour figure can be viewed at wileyonlinelibrary.com]

Each trial began with the presentation of a central fixation cross (white, approximately 1° diameter) for 500 ms, followed by a memory array (Figure 2A). This memory array consisted of either three or five arrows displayed for a period of 1,000 ms, followed by a black screen of 4,000 ms. At the end of each sequence, recall for one of the items was tested. In the standard ‘arrow probe’ condition, the probe constituted a randomly orientated arrow of the same colour as the target item, at the centre of the screen. Participants were instructed to rotate the probe using a computer mouse to match the remembered orientation of the item of the same colour. The mouse cursor began at the centre of the screen, and when the mouse was moved outside a circle radius of 0.7°, the arrow orientation changed to point in the direction of the mouse movement (i.e., the direction from the centre of screen to the mouse coordinate). In the novel ‘dot probe’ condition, initially a dot of the target item's colour (1.3° diameter) was shown at the centre of the screen, instead of the arrow. Once the mouse was moved, this dot *changed into* the arrow probe, making it possible to point the arrow in the intended direction just as in the arrow probe condition.

Each of the participants performed four practice trials consisting of the four different conditions presented in the experiment. For explanatory purposes, these practice trials were significantly longer than the real trials. After this practice phase, participants performed four blocks of 124 trials each. Each block consisted of 31 trials for each of the four possible conditions mentioned above (2 probe type × 2 number of items presented). Trial types were present in equal numbers, and their order was shuffled within each block.

### Experiment 2: Mask condition

#### Stimuli

As in Experiment 1, the memory array consisted of three differently coloured arrows, positioned just as in the first Experiment's three‐item condition.

#### Procedure

**Figure 3:**
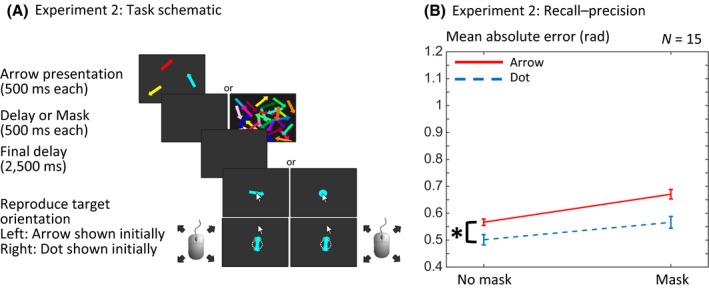
Design and mean absolute error for Experiment 2. (A) Three arrows were presented in the memory array and either followed by a mask of 1,000 randomly orientated and coloured arrows or a black delay screen. The probe could be a coloured arrow or dot, just as in Experiment 1. (B) Replicating the interference effect observed in Experiment 1, we found that recall was worse with a compound probe (*p* = .003). Masking also significantly impaired recall–precision (*p* = .002). But crucially, there was no interaction between the probe type and masking. The error bars were calculated by subtracting each subject's grand mean away from their individual per‐condition values and showing ± the standard error (Loftus & Masson, 1994). [Colour figure can be viewed at wileyonlinelibrary.com]

Fixation period and memory array were identical to the first experiment, followed either by a black screen or a ‘mask’ of 1,000 randomly oriented, randomly coloured, and randomly positioned arrows, which were in both cases presented for 500 ms and followed by a black screen of 3,500 ms (Figure 3A). At the end of each sequence, recall for one of the items was tested as per Experiment 1; again, the probe was either an arrow or initially a dot of the target colour at the centre of the screen that changed to an arrow when the mouse is moved. As per Experiment 1, participants performed four practice trials for explanatory purposes. After this practice phase, participants performed four blocks of 124 trials each. Each block consisted of 31 trials for each of the four possible conditions mentioned above (2 probe type × 2 mask/no mask). The order of different possible trials was shuffled randomly within each block.

### Experiment 3: Sequential presentation

#### Stimuli

Stimuli were presented at a viewing distance and on a monitor as per Experiments 1 and 2. Each memory array consisted of four sequentially presented arrows of different colours (approximately 2.5° × 0.4° of visual angle) presented on a black background. The arrows appeared at random equally spaced locations on the boundary of an invisible circle (radius 6.5°) around fixation. Colours and orientations were chosen randomly as per Experiments 1 and 2.

#### Procedure

**Figure 4:**
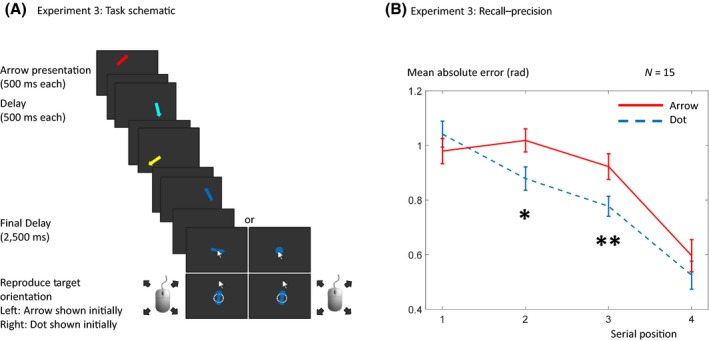
Design and mean absolute error for Experiment 3. (A) Four coloured arrows were sequentially presented in the memory array. The probe could be a coloured arrow or dot, as previously described. (B) There was a significant interaction between the probe type and the serial position of the target item in the sequence (*F*(3,39) = 5.19, *p* = .004). Pairwise tests indicated that recall error was greater for the arrow probe only for the second (*p* = .016) and third (*p* = .004) serial positions, but not for the first (*p* = .26) or last item (*p* = .31) in the sequence. The error bars were calculated by subtracting each subject's grand mean away from their individual per‐condition values and showing ± the standard error (Loftus & Masson, 1994). [Colour figure can be viewed at wileyonlinelibrary.com]

A fixation period as per Experiments 1 was followed by a memory array. This memory array consisted of four sequentially shown arrows, displayed for a period of 500 ms each, and a black screen for a period of 500 ms after each, except for the last arrow which was followed by a 2,500 ms black screen. At the end of the sequence, recall for one of the items was tested by displaying a probe as per Experiments 1 and 2 (Figure 4A).

Each of the participants performed eight practice trials consisting of the eight different conditions presented in the experiment. For explanatory purposes, these practice trials were again significantly longer than the real trials. After this practice phase, participants performed four blocks of 72 trials each. Each block consisted of nine trials for each of the eight possible conditions mentioned above (2 probe type × 4 target position in sequence). The order of different possible trials was random throughout each block. Participants were able to decide whether they wanted to have a break and how long they wanted to pause after each block.

### Precision calculation

We defined the absolute error in recall as the unsigned difference in response angle from target angle. Response precision (the reciprocal of the circular *SD* of errors) is approximately proportional to the reciprocal of the absolute error. The mean absolute error was compared between conditions using two‐way repeated‐measures ANOVA. All trials with subject's response times of 5 s and over were excluded for further analysis as these could be identified as outliers (mean + three times standard deviation, Figure S1). Across all conditions, this criterion excluded an average of 1.22% (*SD* = 2.23%) of trials per subject in Experiment 1, 0.93% per subject (*SD* = 1.38%) in Experiment 2, and 3.96% (*SD* = 4.76%) per subject in Experiment 3. We reran the analysis without this exclusion criterion for the main paradigms and were able to reproduce the results (Table S1).

To model sources of error, we used a Bayesian hierarchical model of the mixture of von Mises model (Bays, Catalao, & Husain, 2009) in R and JAGS as presented by Oberauer, Stoneking, Wabersich, and Lin (2017) to estimate the relative contribution of precision, target response, non‐target response, and guessing. Non‐target responses are ‘swap errors’ in which participants report the orientation of one of the other, unprobed items in the memory array. Random‐guessing responses are selected from a uniform distribution. For this purpose, 2,500 adaptations, 5,000 iterations, and four chains were used.

## Results

### Experiment 1: Simultaneous presentation

To test whether the mean absolute error was worsened by probe interference, a 2 × 2 ANOVA of probe type × set size was performed. There was an overall significant main effect of probe type, that is, of whether the memory probe started off as an arrow or was initially just a dot (Figure 2B, *F*(1,14) = 5.88, *p* = .029, $\eta_{p}^{2}$ = 0.296). Thus, recall–precision was significantly lower with an arrow probe. As expected, there was also a main effect of number of items presented (*F*(1,14) = 127.22, *p* < .001, $\eta_{p}^{2}$ = 0.901). There was no interaction between set size and probe type (*F*(1,14) = 0.58, *p* = .459, $\eta_{p}^{2}$ = 0.040). A JZS Bayes factor ANOVA (Love *et al*., 2015; Morey & Rouder, 2015; Rouder, Morey, Speckman, & Province, 2012) with default prior scales revealed that in absolute error, the model with both main effects (BF_10_ = 3.819e+16) was preferred to the interaction model (BF_10_ = 1.344e+16) by a Bayes factor of 2.84 and to the probe type‐only model (BF_10_ = 0.323) by a Bayes factor of 1.18e+17. The set size‐only model (BF_10_ = 3.840e+16) was preferred by a Bayes factor of 1.01 to the main effect model (Table S2).

**Figure 5:**
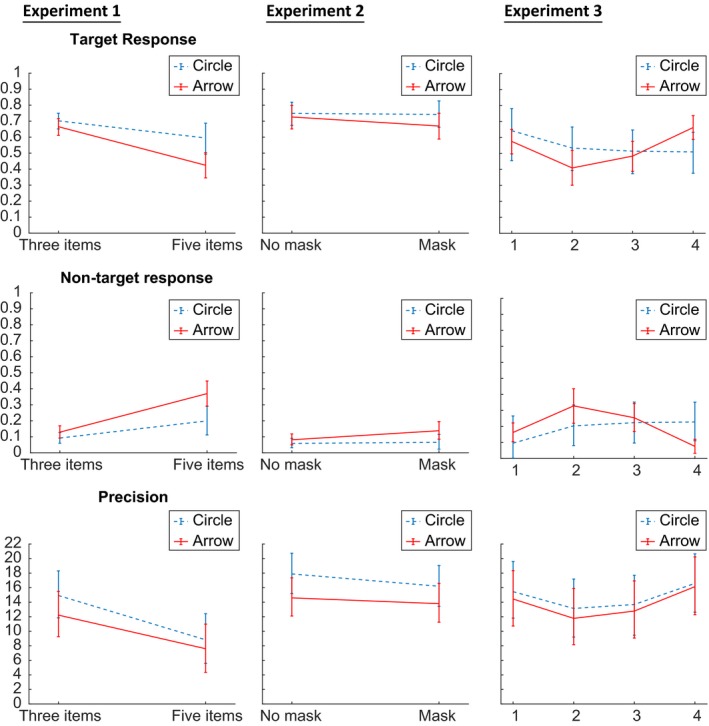
Plots of Bayesian hierarchical model fits for Experiments 1–3 (left to right). Plots for target response in the first line, for non‐target responses in the second line, for guessing responses in the third line, and for precision in the bottom line. Error bars represent highest density interval from a sample of representative values, estimated as shortest credible interval (Oberauer *et al*., 2017). [Colour figure can be viewed at wileyonlinelibrary.com]

We compared models that allowed either set size, probe type, both factors (and their potential interaction) or neither of these two factors to have an effect on the model parameters using the WAIC. For this purpose, priors were set to the same values over the four different models. The results support – similar to the ANOVA – a model of both factors without interaction. We plotted the sources of error and precision for the full interaction model (Figure 5). In the above models, the probe type could influence both precision and probability of reporting the correct target item. In addition, we tested fits for models that allowed probe type and set size to have an effect on guessing. In most cases, the WAIC preferred models that do not allow an effect on guessing. Next, we asked whether a probe type model that only allowed an effect on precision or target response was the best fitting model (Table 1). The comparison of WAICs showed that the precision model was the best fitting model.

**Table 1:** WAIC values of hierarchical Bayesian measurement mixture models for Experiment 1

	Effects allowed on target response and precision	Effects allowed on target response, guessing response, and precision
No effect	13,618.380	13,607.520
Probe effect only	13,543.921	13,549.762
Set size effect only	13,283.385	13,393.291
Both effects	13,248.453^a^	13,370.171
Both effects and interaction	13,250.460	13,353.313^a^
Probe effect only, on precision only	13,550.639†	13,543.791†
Probe effect only, on target response only	13,565.585	13,552.556

*Notes*

a Best model fit main analysis.

† Best model fit subanalysis.

We also investigated whether the type of probe influenced the time to begin moving the mouse, from the moment the probe was presented on the screen (Figure S2). A 2 × 2 ANOVA of number of items × probe type showed that there was no significant difference in the time to initiate responses (*F*(1,14) = 1.76; *p* = .21; $\eta_{p}^{2}$ = 0.112), between arrow and dot conditions. As expected, there responding was slower for the 5‐item condition (*F*(1,14) = 14.54; *p* = .002; $\eta_{p}^{2}$ = 0.509) but with no interaction (*F*(1,14) = 0.05; *p* = .83; $\eta_{p}^{2}$ = 0.004). The Bayes factor ANOVA for the time to initiate responses discovered that the set size‐only model (BF_10_ = 4004.63) was preferred to the probe type‐only model (BF_10_ = 0.369) by a Bayes factor of 1.0853e+04, to the model with both main effects (BF_10_ = 1769.87) by a Bayes factor of 2.26, and to the interaction model (BF_10_ = 662.40) by a Bayes factor of 6.05 (Table S3).

One further way in which the probe may drive errors is if the initial probe angle biases responses. To look for such an effect, we plotted the signed angular error, that is, the difference between target and response angle, against the difference between initial probe angle and target angle (Figure S3). To do this, we examined only arrow probe trials, collapsed across the other conditions in each experiment. The quantiles of the response error are shown as coloured lines, calculated in sliding window bins of width 20% over the possible relative probe orientations. Possible theoretical patterns of probe interference are (1) increased interference when probe is similar to the target, (2) increased interference when probe is dissimilar to the target, (3) tendency to report angle closer to probe, or (4) tendency to report angle as further away from probe's orientation (Figure S3A). But rather than following any of these patterns, interference from the probe appeared to be independent of the probe's orientation (Figure S3B).

### Experiment 2: Masking

For the second experiment, we performed a 2 × 2 ANOVA of probe type × mask on mean absolute error. The results of Experiment 1 were replicated, with a significant main effect of probe type (*F*(1,14) = 14.66, *p* = .002; $\eta_{p}^{2}$ = 0.511). The presence of the mask significantly increased error (main effect of mask vs. no‐mask, *F*(1,14) = 16.51, *p* = .001; $\eta_{p}^{2}$ = 0.541) suggesting that the mask effectively erased some visual information, making it significantly more difficult for participants to reproduce the arrow correctly. However, the mask did not abolish the effect of probe type, indicated by no interaction between probe type and masking (*F*(1,14) = 0.98; *p* = .338; $\eta_{p}^{2}$ = 0.066). Specifically, recall was worse for the arrow probe than the dot probe, both in the masked (*t*(14) = 2.66, *p* = .019) and in the no‐mask conditions (*t*(14) = 3.06, *p* = .008; Figure 3B). A Bayes factor ANOVA for the absolute error demonstrated that the model with both main effects (BF_10_ = 2522.73) was preferred to the masking‐only model (BF_10_ = 26.62) by a Bayes factor of 94.77, to the probe type‐only model (BF10 = 26.61) by a Bayes factor of 94.80, and to the interaction model (BF_10_ = 1322.103) by a Bayes factor of 1.91 (Table S4).

We performed the same hierarchical Bayesian models as in Experiment 1, comparing model fits if either probe type, masking, both (and their potential interaction) or none of the factors were allowed to have an effect on the modelling. The comparison of WAIC again – similar to the results of the ANOVA – prefers a model of both effects, probe type, and masking (Table 2). In most cases, the WAIC preferred models that do not allow an effect on guessing. And again, we plotted the sources of error and precision for the full interaction model (Figure 5). A subanalysis of a probe type model that allowed either an effect on precision or target response showed a preference for the model that allowed an effect on precision.

**Table 2:** WAIC values of hierarchical Bayesian measurement mixture models for Experiment 2

	Effects allowed on target response and precision	Effects allowed on target response, guessing response, and precision
No effects	7,910.483	7,905.507
Probe effect only	7,851.580	7,850.399^a^
Mask effect only	7,856.709	7,863.681
Both effects	7,838.427^a^	7,853.540
Both effects and interaction	7,843.414	7,850.881
Probe effect only, on precision only	7,842.153†	7,851.524†
Probe effect only, on target response only	7,863.517	7,868.239

*Notes*

a Best model fit main analysis.

† Best model fit subanalysis.

As in Experiment 1, there was no difference in the time to begin (*F*(1,14) = 0.77; *p* = .39; $\eta_{p}^{2}$ = 0.052) responses between arrow and dot conditions, nor mask versus no‐mask conditions (*F*(1,14) = 0.90; 0.36; $\eta_{p}^{2}$ = 0.052), nor was there an interaction (*F*(1,14) = 1.46; *p* = .25; $\eta_{p}^{2}$ = 0.095). The Bayes factor analysis discovered that for the time to initiate responses, the null model was preferred to the masking‐only model (BF_10_ = 0.35) by a Bayes factor of 2.90, to the probe type‐only model (BF_10_ = 0.44) by a Bayes factor of 2.2831, to the model with both main effects (BF_10_ = 0.16) by a Bayes factor of 6.41, and to the interaction model (BF_10_ = 0.07) by a Bayes factor of 13.70 (Table S5).

Again, there was no indication that the initial probe angle biased responses (Figure S3C).

In order to provide better power to detect effects of probe type on sources of error, we pooled data across Experiments 1 and 2. The combined analysis indicates that the probe interference has effects on guessing as well as target responses and precision (Table S9).

### Experiment 3: Sequential presentation

For the mean absolute error, we performed a 2 × 4 ANOVA of probe type × serial position. This showed a significant interaction between probe type and position of the target item in the sequence (*F*(3,42) = 3.83; *p* = .016; $\eta_{p}^{2}$ = 0.215). As expected, there was also a main effect of the position of the target item in the sequence (*F*(3,42) = 21.83, *p* < .001; $\eta_{p}^{2}$ = 0.609) but not of the probe type (*F*(3,42) = 3.44, *p* = .085; $\eta_{p}^{2}$ = 0.197). Pairwise *t*‐tests demonstrated that recall error in the arrow probe was only higher in the second (*t*(14) = 2.205, *p* = .045) and third (*t*(14) = 3.383, *p* = .004) but not in the first (*t*(14) = 0.963, *p* = .352) and last serial positions (*t*(14) = 1.195, *p* = .252; Figure 4B). The Bayes factor analysis showed that for absolute error, the model with both main effects (BF_10_ = 1.093e+11) is preferred to the interaction model (BF_10_ = 5.941e+10) by a Bayes factor of 1.84, to the sequence‐only model (BF_10_ = 9.140e+10) by a Bayes factor of 1.20, and to the probe type‐only model (BF_10_ = 0.524) by a Bayes factor of 2.0859e+11 (Table S6).

In addition, we performed *post‐hoc* comparisons between the absolute error in different sequential positions for both probe types (Table S7). These show a strong tendency for error being higher in the earlier presented items compared to the later presented items for both probe types. The lack of effect for the first item could not be explained by performance being at chance, that is, a floor effect (Kolmogorov–Smirnov test for responses to first item vs. chance, *p* < .001 for both arrow and dot probe; Figure S4).

As per Experiments 1 and 2, we used the comparison between the different hierarchical model fits allowing probe type, sequence position, both of them (and their interaction) and none of those to have an effect on the model parameters. The WAIC preferred a model including only an effect of sequential position, closely followed by the ‘both effects’ and ‘interaction’ models (Table 3). In most cases, the WAIC preferred models that do not allow any effects on guessing. Sources of error and precision are plotted separately for the full interaction models (Figure 5).

**Table 3:** WAIC values of hierarchical Bayesian measurement models for Experiment 3

	Effects allowed on target response and precision	Effects allowed on target response, guessing response, and precision
No effect	7,487.645	7,488.79
Probe effect only	7,432.999	7,426.596
Sequence effect only	7,331.279	7,386.903
Both effects	7,339.379	7,386.408^a^
Both effects and interaction	7,326.635^a^	7,405.406
Probe effect only, on precision only	7,437.266	7,425.68†
Probe effect only, on target response only	7,435.024†	7,426.272

*Notes*

a Best model fit main analysis.

† Best model fit subanalysis.

According to our proposed pattern‐completion hypothesis, responses should take longer for items at positions one to three (which must be retrieved) compared to the last item (which is in the focus of attention). Using a standard ANOVA, there was no main effect of probe type (*F*(1,42) = 0.021; *p* = .886; $\eta_{p}^{2}$ = 0.002) or sequence position (*F*(3,42) = 2.11; *p* = .113; $\eta_{p}^{2}$ = 0.131) on the time to begin responses. There was also no interaction of these two (*F*(3,42) = 2.722; *p* = .056; $\eta_{p}^{2}$ = 0.163). A Bayes factor ANOVA for the time to initiate responses preferred the null model to the sequence‐only model (BF_10_ = 0.84) by a Bayes factor of 1.19, to the probe type‐only model (BF_10_ = 0.20) by a Bayes factor of 5.08, to the model with both main effects (BF_10_ = 0.16) by a Bayes factor of 6.17, and to the interaction model (BF_10_ = 0.07) by a Bayes factor of 14.71 (Table S8).

## Discussion

The experiments reported here aimed to test whether irrelevant features presented as part of the memory probe, at the time of retrieval, are detrimental to recall. We compared performance in the case where the probe initially contained a feature on the dimension that had to be recalled (coloured arrow probe), with a case where there was initially no information on the feature dimension to be recalled (coloured dot probe). The results of our first experiment showed that the presence of this feature significantly impaired participants’ precision, as they performed significantly better when the probe was initially a colour‐only dot (Figures 2B, 3B, and 4B).

Many methods of measuring WM involve presenting a probe that contains a feature on the dimension to be recalled that is not the correct feature value to be recalled. For example, adjustment tasks involve a probe that already holds an orientation, which may or may not match the target. The method of adjustment allows measurement of the precision of recall, but may corrupt retrieval of the recalled object by interference from the probe. This impairment could be significantly decreased by using a colour‐only probe, in which the probe is initially just a coloured dot. Importantly, the probe type did not influence the reaction time to begin the motor response, indicating that even though retrieval was improved with the colour‐only probe, it cannot be accounted for by a longer response time – for example, a speed–accuracy trade‐off or less impulsive responding.

In Experiment 2, we asked whether the observed effects arise because the irrelevant feature in the probe overwrites perceptual information. If there were a visual immediate buffer that holds additional information about the most recently presented visual information, such as an iconic store, interference from the additional probe feature might overwrite the additional immediate buffer. Although both probe interference and the addition of a mask significantly reduced precision of recall, there was no interaction, suggesting that the degree of interference by the probe was not attenuated by the introduction of a mask. Assuming that the mask disrupted any purely perceptual, iconic buffer, then the arrow probe must degrade performance by a different mechanism.

According to some models of WM, probes trigger retrieval by reactivating the features of corresponding items in memory (Farrell & Lewandowsky, 2002; Sandberg, Tegnér, & Lansner, 2003). One item in memory may already be in the focus of attention, which means that its features are already active, in a response‐ready state. Items that are not currently in the focus of attention at the time of recall must first be reactivated by the probe, in order to generate a response. One example of an attentional focus may be the recency effect, in which the last item in a sequence is recalled better, perhaps because its features are held in an active state (Blalock & Clegg, 2010; Gorgoraptis *et al*., 2011; Hay, Smyth, Hitch, & Horton, 2007; Zokaei *et al*., 2011). In this case, interference from the irrelevant probe features would be expected to impede recall only when the item must be reactivated from a non‐focused state, that is, for non‐final items. Therefore, we conducted the third experiment in which we presented a set of four items in a sequence to participants.

The results showed that the last item was immune to disruption by the arrow. However, the second and third items were not. This suggests that the presence of a compound probe selectively disrupts the focusing of earlier items in the sequence. Items that were recalled better due to recency effect, however, were not influenced by probe interference. Thus, we saw a significant interaction of probe type and position of the target item in the sequence. Several authors have considered whether this most recent item might automatically be held in the focus of attention. An associative pattern completion model predicts worse recall accuracy for unfocused items, when an irrelevant feature on the dimension to be recalled is present in the probe (Manohar *et al*., 2017). This is because they have to be brought into the focus of attention by reactivation, and this process is subject to interference.

This idea is broadly in line with data presented here. However, it does not explain why the first item in the sequence is also immune to the effect. Primacy effects in visuospatial WM tend to be small and their mechanism is poorly understood, but may involve association between the first item and the preceding context, as proposed for episodic memories, perhaps relying on the medial temporal lobe. An alternative explanation of probe interference in general could be that the inner items in a sequence are at least partly held in a different kind of store, for example, in episodic memory, which is harder to retrieve from (Oztekin, McElree, Staresina, & Davachi, 2009; Oztekin *et al*., 2010). It is, however, difficult to determine whether long‐term memory is involved in the case of the first item. The lack of probe interference for the first item in the sequence is unlikely to be a floor effect, because performance for the first item is still better than chance (Figure S4).

When sources of error in the Bayesian hierarchical approach for the mixture model were estimated, model comparison indicated an independent main effect of interference in the first and second experiments (Tables 1 and 2). The computational modelling permitted us to distinguish between possible mechanisms of interference, in addition to simply quantifying the errors caused by interference. Estimates of the sources of error (Figure 5) suggest that the arrow probe both increased misbinding to non‐target stimuli and decreased response precision, independently of masking, and amplified by larger set size.

In the third experiment, the interference is manifest in reduced precision (Table 3), with (Figure 5) misbinding being reduced especially in the second sequential position.

According to the pattern‐completion hypothesis, we expected that responses in the last experiment should take longer for items at positions one to three (which must be retrieved) compared to the last item (which is in the focus of attention). Our data did not, however, support this (Figure S2). There was only a trend towards an interaction (*p* = .056, not supported by Bayesian ANOVA). Without interference, the time to initiate responses is relatively independent of serial position, but the probe interference may selectively slow retrieval for ‘middle’ items in the sequence. Possible reasons for the lack of effect could be that the task permits adjustment, so participants may move the mouse before completing retrieval. Another possibility is that probe interference increases retrieval error without slowing the process of pattern completion itself – this possibility is supported by the increase in misbinding errors, which in the model would comprise erroneous completions of a valid memory item. It is also possible that our sample size might not be adequately powered to reveal small changes in reaction time. Therefore, further studies would help confirm the presence of the model‐predicted reaction time effects.

Our task required participants to simply move the mouse in the direction of the remembered arrow. Comparing with other tasks, it is important to distinguish carefully between tasks in which a decision must be made based on the probe stimulus and tasks which require simply reporting items in memory. If information at the time of probe must interact with corresponding information in the memory item, for example, in change detection, comparisons with memory, or in recall‐by‐adjustment paradigms, then we should consider those tasks as involving more than just memory recall. They additionally involve a decision process, and interference at recall may manifest in the decision‐making components of these tasks. Comparing these more complex paradigms to simple report, with and without interference, may enable separation of these recall and decision‐making components (Myers, Stokes, & Nobre, 2017).

Task sets themselves are likely to involve WM, especially in complex tasks (e.g., Schumacher *et al*., 2018). Conflict between task rules (e.g., as seen in the Simon task) might thus involve similar mechanisms to those involved in WM retrieval. In particular, task rules can be viewed as an associative mapping between stimulus and response – in the same way as WM binding between features of an object. On this view, selecting a response in response conflict tasks involves matching a cue to the appropriate rule in memory. Response selection in response to a cue can be considered as a motor analogue of recalling a memory item in response to a probe. Conversely, WM can be regarded as a ‘ready‐state’ for selecting and reactivating recently encountered information (Myers *et al*., 2017). Specifically, if choosing an appropriate action after a cue involves a kind of pattern completion, it should be subject to the interference effects similar to probe effects studied here. Indeed, response conflict correlates with WM capacity (Duncan *et al*., 2008), with costs arising from interference between task representations, rather than simply from additional stimuli or responses (Schumacher *et al*., 2018). This supports the idea that task representations themselves are cognitively costly and may at least partly tax WM. Such an equivalence between the processes of executing task rules and recalling items in WM would generate a range of testable predictions for future studies.

Our results, consistent with the conclusions of Souza *et al*. (2016), demonstrate that the method of probing can itself impact on WM precision, and this interference is likely to be relevant to many previous studies that use well‐established probing methods. The pattern of interference is consistent with a pattern‐completion account of recall. The recency effect provides resilience to this interference, as predicted by accounts of the focus of attention. These findings are in keeping with an account in which visual WM representations are content addressable, associative, and modulated by attention, with interference playing a crucial role in forgetting.

In conclusion, our findings suggest that the probe in WM experiments can interfere with recall, and the process of retrieval is modulated by a number of factors including the focus of attention.

## Supporting information


**Figure S1.** Histograms of the period from moment the probe appears on the screen to finishing the response in experiments 1 (A) and 2 (B).
**Figure S2.** Times to initiate responses from the moment, the probe appears on the screen.
**Figure S3.** Does the orientation of the probe arrow influence the response?.
**Figure S4.** Empirical distribution function of signed error in responses for the first sequentially presented item in experiment 3.

**Supplementary Materials.** Merged datasets confirm strong probe‐type effects for all sources of error.
**Table S1.** Experimental data without exclusion criterion.
**Table S2.** Bayes factor ANOVA for the Absolute Error in Experiment 1.
**Table S3.** Bayes factor ANOVA for the Time to initiate responses in Experiment 1.
**Table S4** Bayes factor ANOVA for the Absolute Error in Experiment 2.
**Table S5.** Bayes factor ANOVA for the Time to Initiate Responses in Experiment 2.
**Table S6.** Bayes factor ANOVA for the Absolute Error in Experiment 3.
**Table S7.** *Post‐hoc* T‐Tests on absolute error by sequence position for Experiment 3.
**Table S8.** Bayes factor ANOVA for the Time to Initiate Responses in Experiment 3.
