# Supplementary material for: Recall cues interfere with retrieval from visuospatial working memory
Source: Br J Psychol. 2019 Jan 2;110(2):288–305. doi: 10.1111/bjop.12374 (PMC6590415; doi:10.1111/bjop.12374)
Supplement: Supplementary file 1 — Figure S1. Histograms of the period from moment the probe appears on the screen to finishing the response in experiments 1 (A) and 2 (B). Figure S2. Times to initiate responses from the moment, the probe appears on the screen. Figure S3. Does the orientation of the probe arrow influence the response?. Figure S4. Empirical distribution function of signed error in responses for the first sequentially presented item in experiment 3. [file BJOP-110-288-s001.pptx]

## Slide 1
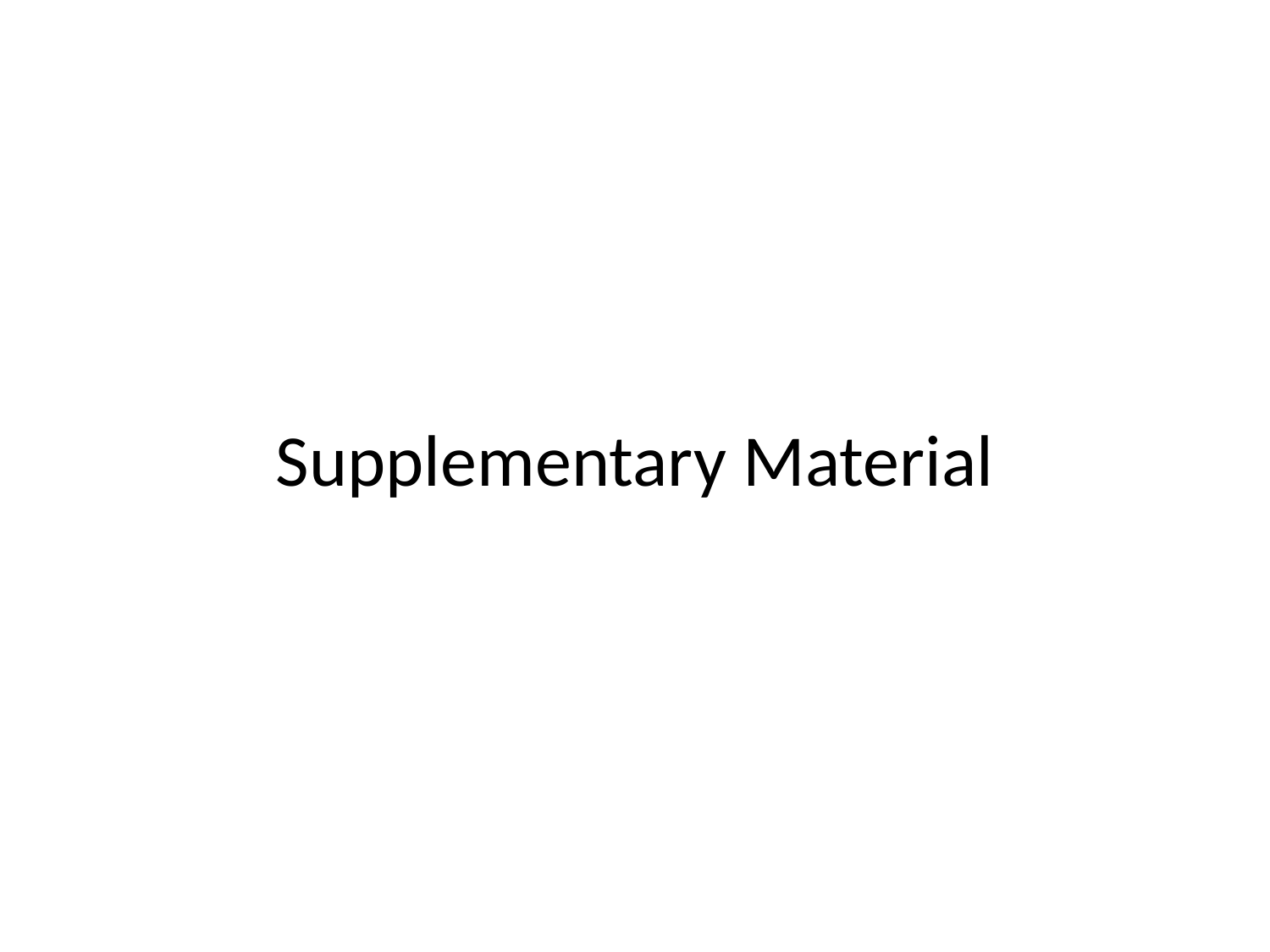

# Supplementary Material

## Slide 2
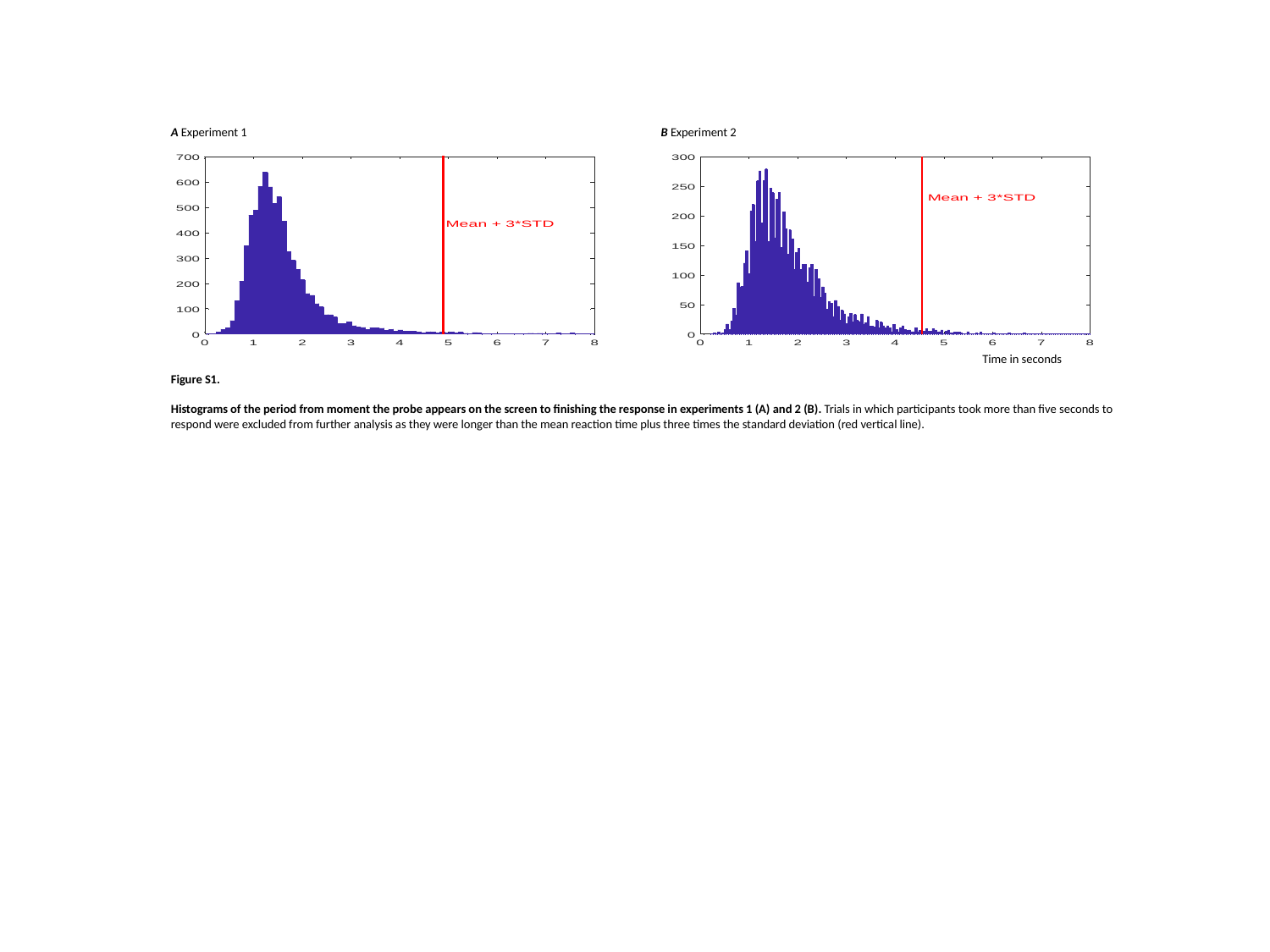

A Experiment 1
B Experiment 2
Time in seconds
Figure S1.
Histograms of the period from moment the probe appears on the screen to finishing the response in experiments 1 (A) and 2 (B). Trials in which participants took more than five seconds to respond were excluded from further analysis as they were longer than the mean reaction time plus three times the standard deviation (red vertical line).

## Slide 3
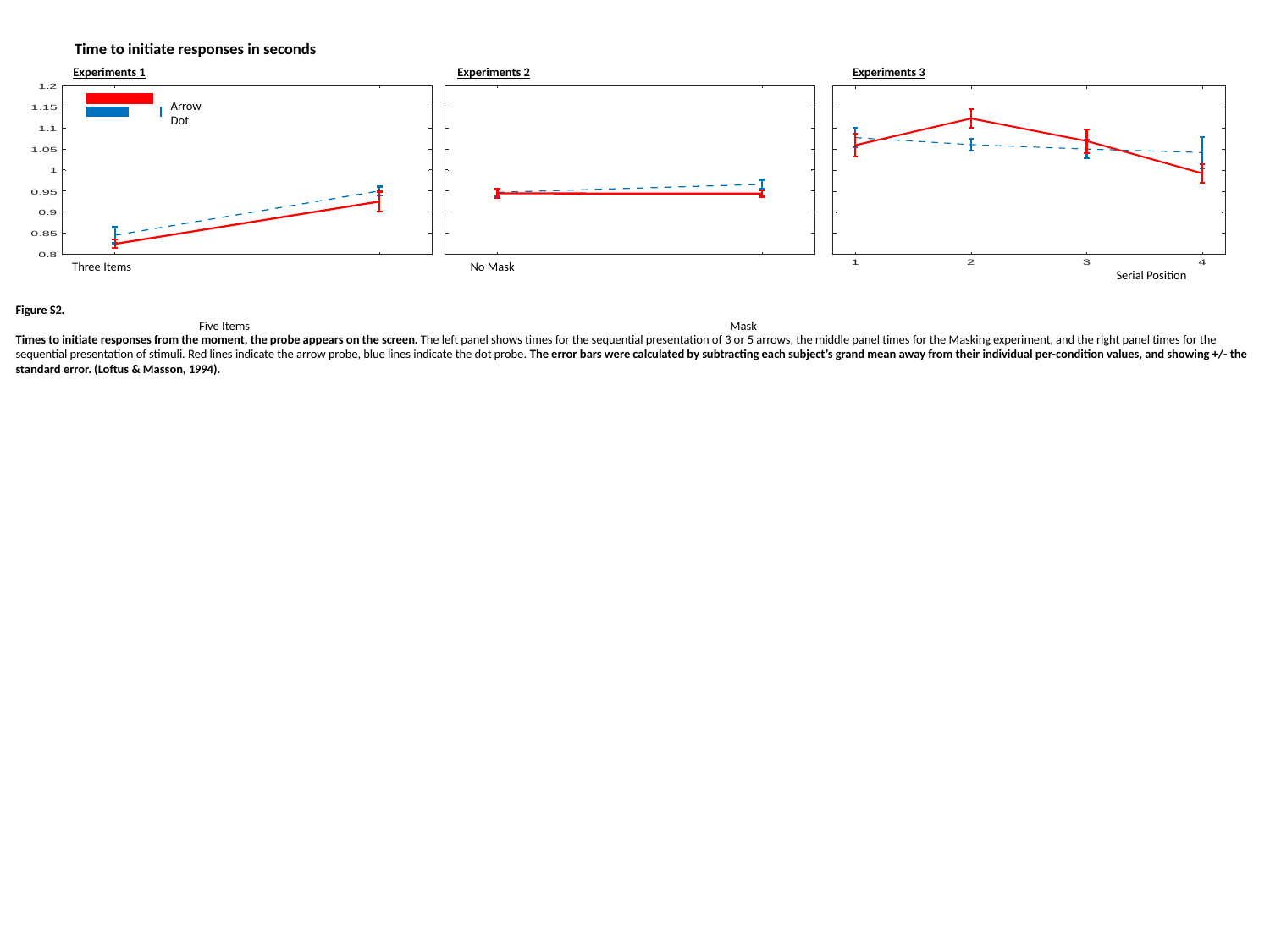

Time to initiate responses in seconds
Experiments 1
Experiments 2
Experiments 3
Arrow
Dot
Three Items									Five Items
No Mask										 Mask
Serial Position
Figure S2.
Times to initiate responses from the moment, the probe appears on the screen. The left panel shows times for the sequential presentation of 3 or 5 arrows, the middle panel times for the Masking experiment, and the right panel times for the sequential presentation of stimuli. Red lines indicate the arrow probe, blue lines indicate the dot probe. The error bars were calculated by subtracting each subject’s grand mean away from their individual per-condition values, and showing +/- the standard error. (Loftus & Masson, 1994).

## Slide 4
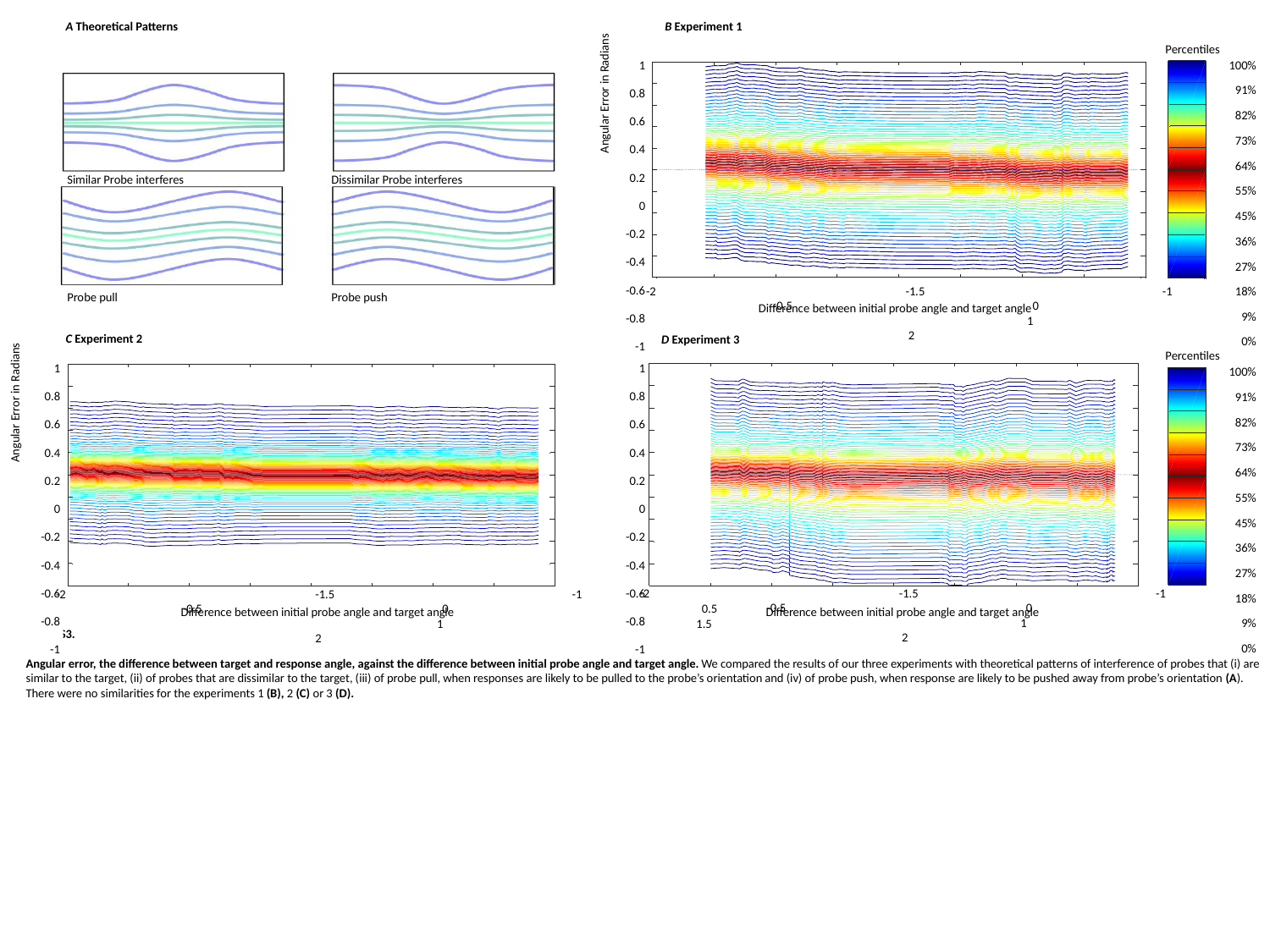

A Theoretical Patterns
B Experiment 1
Percentiles
1
0.8
0.6
0.4
0.2
0
-0.2
-0.4
-0.6
-0.8
-1
100%
91%
82%
73%
64%
55%
45%
36%
27%
18%
9%
0%
Angular Error in Radians
Similar Probe interferes
Dissimilar Probe interferes
-2		 -1.5		 -1		-0.5		 0		 0.5			1		 1.5		 2
Probe pull
Probe push
Difference between initial probe angle and target angle
C Experiment 2
D Experiment 3
Percentiles
1
0.8
0.6
0.4
0.2
0
-0.2
-0.4
-0.6
-0.8
-1
1
0.8
0.6
0.4
0.2
0
-0.2
-0.4
-0.6
-0.8
-1
100%
91%
82%
73%
64%
55%
45%
36%
27%
18%
9%
0%
Angular Error in Radians
-2		 -1.5		 -1		-0.5		 0		 0.5			1		 1.5		 2
-2		 -1.5		 -1		-0.5		 0		 0.5			1		 1.5		 2
Difference between initial probe angle and target angle
Difference between initial probe angle and target angle
Figure S3.
Angular error, the difference between target and response angle, against the difference between initial probe angle and target angle. We compared the results of our three experiments with theoretical patterns of interference of probes that (i) are similar to the target, (ii) of probes that are dissimilar to the target, (iii) of probe pull, when responses are likely to be pulled to the probe’s orientation and (iv) of probe push, when response are likely to be pushed away from probe’s orientation (A). There were no similarities for the experiments 1 (B), 2 (C) or 3 (D).

## Slide 5
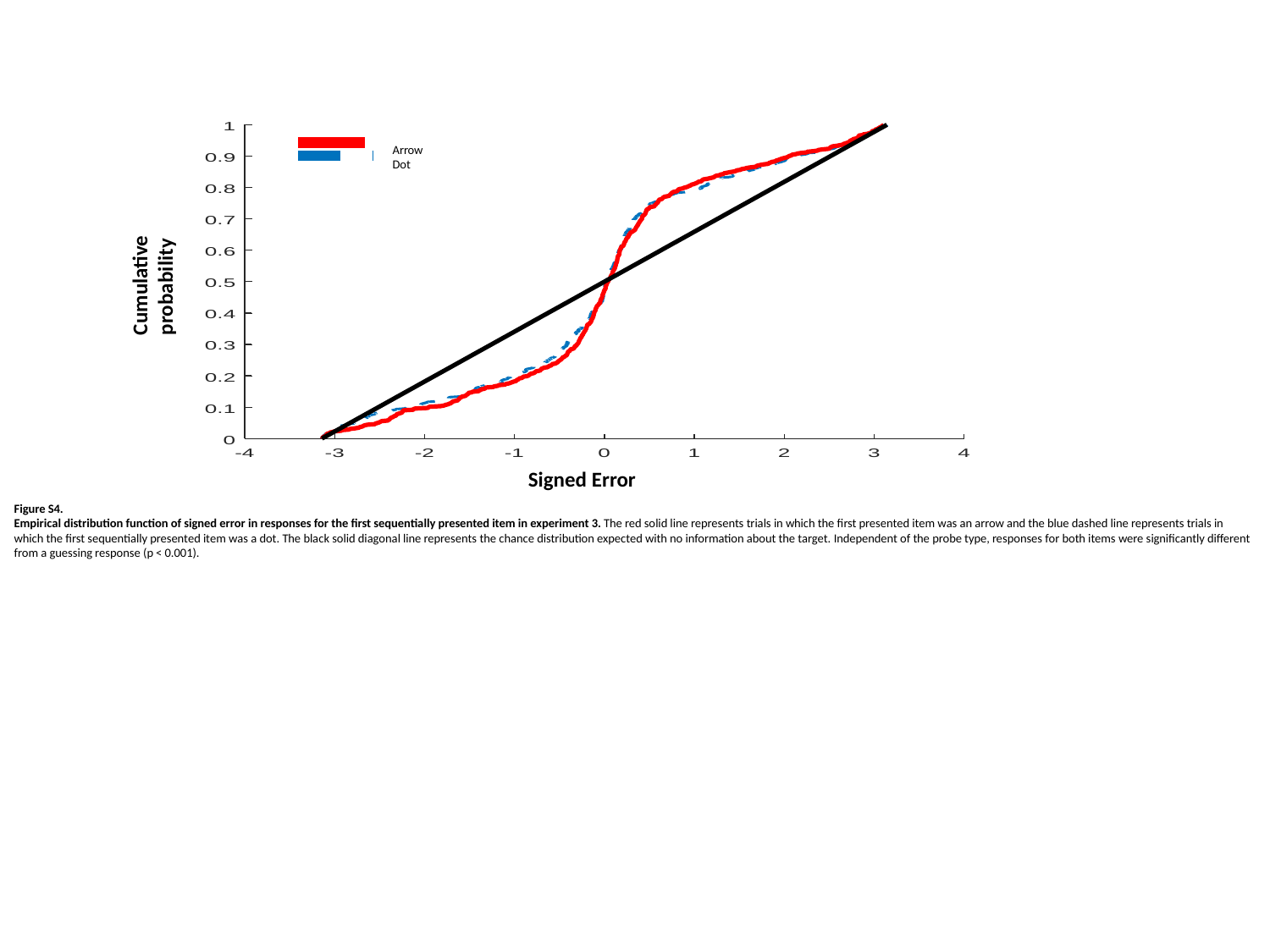

Arrow
Dot
Cumulative probability
Signed Error
Figure S4.
Empirical distribution function of signed error in responses for the first sequentially presented item in experiment 3. The red solid line represents trials in which the first presented item was an arrow and the blue dashed line represents trials in which the first sequentially presented item was a dot. The black solid diagonal line represents the chance distribution expected with no information about the target. Independent of the probe type, responses for both items were significantly different from a guessing response (p < 0.001).
