# Supplementary material for: Recall cues interfere with retrieval from visuospatial working memory
Source: Br J Psychol. 2019 Jan 2;110(2):288–305. doi: 10.1111/bjop.12374 (PMC6590415; doi:10.1111/bjop.12374)
Supplement: Supplementary file 2 — Supplementary Materials. Merged datasets confirm strong probe‐type effects for all sources of error. Table S1. Experimental data without exclusion criterion. Table S2. Bayes factor ANOVA for the Absolute Error in Experiment 1. Table S3. Bayes factor ANOVA for the Time to initiate responses in Experiment 1. Table S4 Bayes factor ANOVA for the Absolute Error in Experiment 2. Table S5. Bayes factor ANOVA for the Time to Initiate Responses in Experiment 2. Table S6. Bayes factor ANOVA for the Absolute Error in Experiment 3. Table S7. Post‐hoc T‐Tests on absolute error by sequence position for Experiment 3. Table S8. Bayes factor ANOVA for the Time to Initiate Responses in Experiment 3. [file BJOP-110-288-s002.docx]

**Supplementary Materials**

**Merged datasets confirm strong probe-type effects for all sources of error**

In addition to the analysis presented above, we pooled trials from the “three items, dot vs. arrow”-condition of Experiment 1 and of the “no mask, dot vs. arrow”-condition of Experiment 2. Taking absolute error of this combined dataset, we performed a repeated measures ANOVA with repeated measures on probe type and the between-subject factor “group”. Results showed a strongly significant main effect of probe type (F(1,28) = 15.55, p < 0.001, η_p^2 = 0.357), but no interaction of group and probe type (F(1,28) = 0.007, p = 0.934, η_p^2 = 0.000). Concerning between-subjects effects, there was also no significant main effect of group (F(1,28) = 1.38, p = 0.251, η_p^2 = 0.047). A Bayesian repeated measures ANOVA preferred the probe type only model (BF10 = 60.23) to the model that included both main effects (BF10 = 48.60) by a Bayes factor of 1.24, to the interaction model (BF10 = 16.509) by a Bayes factor of 3.65 and to the group only model (BF10 = 0.731) by a Bayes factor of 82.39. We also calculated the same Bayesian hierarchical model estimates as per experiments 1-3 (Supplementary Table 10). Again, models that account for the probe type presented with the best model fit when WAICs were compared. The effect seems to especially effect working memory precision.

We also merged the same conditions for the pilot data (three items dot vs. arrow and no mask, dot vs arrow) to perform a repeated measures ANOVA on absolute error. Again, there was a strongly significant main effect of probe type (F(1,29) = 13.33, p = 0.001, η_p^2 = 0.307), but no interaction of group and probe type (F(1,29) = 1.121, p = 0.298, η_p^2 = 0.026). Concerning between-subjects effects, there was also no significant main effect of group (F(1,29) = 0.35, p = 0.558, η_p^2 = 0.012). A Bayesian repeated measures ANOVA preferred the probe type only model (BF10 = 26.46) to the model including both main effects (BF10 = 17.31) by a Bayes factor of 1.528, to the interaction model (BF10 = 9.14) by a Bayes factor of 2.90 and to the group only model (BF10 = 0.66) by a Bayes factor of 40.03.

These analyses further support the case of the arrow probe impairing working memory precision, with the probe type effect presenting with a significantly high effect size in both cases.

- **Supplementary Figure Captions**

**Figure S1.**

**Histograms of the period from moment the probe appears on the screen to finishing the response in experiments 1 (A) and 2 (B).**Trials in which participants took more than five seconds to respond were excluded from further analysis as they were longer than the mean reaction time plus three times the standard deviation (red vertical line).

**Figure S2.**

**Times to initiate responses from the moment, the probe appears on the screen.** The left panel shows times for the sequential presentation of 3 or 5 arrows, the middle panel times for the Masking experiment, and the right panel times for the sequential presentation of stimuli. Red lines indicate the arrow probe, blue lines indicate the dot probe. **The error bars were calculated by subtracting each subject’s grand mean away from their individual per-condition values, and showing +/- the standard error. (Loftus & Masson, 1994).**

**Figure S3.**

**Does the orientation of the probe arrow influence the response?** Angular error is plotted as a function of the probe's orientation. Lines represent quantile contours of angular error, i.e. the difference between target and response angle. The values are binned according to the difference between initial probe angle and target angle, on the x-axis, such that negative values indicate that the probe arrow started anticlockwise relative to the target item. **A)** Four possible theoretical patterns of interference in which responses are (i) less precise when the probe is similar to the target, (ii) less precise when probes are dissimilar to the target, (iii) tend to be closer to the probe’s orientation and (iv) repelled by the probe’s orientation **(A).** **(B-D)** There were no apparent effects of the probe's orientation for experiments 1, 2 and 3.

**Figure S4.**

**Empirical distribution function of signed error in responses for the first sequentially presented item in experiment 3.** The red solid line represents trials in which the first presented item was an arrow and the blue dashed line represents trials in which the first sequentially presented item was a dot. The black solid diagonal line represents the chance distribution expected with no information about the target. Independent of the probe type, responses for both items were significantly different from a guessing response (p < 0.001).

- **Supplementary Tables**

**Supplementary Table 1: Experimental data without exclusion criterion**

| **Factor** | **Absolute Error** | |
| --- | --- | --- |
| *Experiment 1* | | |
| **Setsize** | F(1,14) = 132.47 | p < 0.001 |
| **Probe Type** | F(1,14) = 5.65 | p = 0.032 |
| **Interaction** | F(1,14) = 0.675 | p = 0.425 |
| *Experiment 2* | | |
| **Masking** | F(1,14) = 15.23 | p = 0.002 |
| **Probe Type** | F(1,14) = 14.75 | p = 0.002 |
| **Interaction** | F(1,14) = 1.04 | p = 0.326 |
| *Experiment 3* | | |
| **Sequence Position** | F(3,39) = 18.50 | p < 0.001 |
| **Probe Type** | F(1,13) = 7.24 | p = 0.019 |
| **Interaction** | F(3,39) = 4.26 | p = 0.011 |

| Model | P(M) | P(M\|data) | BF_M_ | BF_10_ | error % |
| --- | --- | --- | --- | --- | --- |
| Null model (incl. subject) | 0.200 | 1.111e-17 | 4.443e-17 | 1.000 |  |
| Setsize | 0.200 | 0.427 | 2.975 | 3.840e+16 | 2.355 |
| Probe Type | 0.200 | 3.586e-18 | 1.435e-17 | 0.323 | 1.534 |
| Setsize + Probe Type | 0.200 | 0.424 | 2.947 | 3.819e+16 | 3.037 |
| Setsize + Probe type +  Setsize * Probe Type | 0.200 | 0.149 | 0.702 | 1.344e+16 | 2.434 |

**Supplementary Table 2: *Bayes factor ANOVA for the Absolute Error in Experiment 1***

**Supplementary Table 3: *Bayes factor ANOVA for the Time to initiate responses in Experiment 1***

| Model | P(M) | P(M\|data) | BF_M_ | BF_10_ | error % |
| --- | --- | --- | --- | --- | --- |
| Null model (incl. subject) | 0.200 | 1.553e-4 | 6.214e-4 | 1.000 |  |
| Setsize | 0.200 | 0.622 | 6.582 | 4004.634 | 1.460 |
| Probe Type | 0.200 | 5.730e-5 | 2.292e-4 | 0.369 | 1.079 |
| Setsize + Probe Type | 0.200 | 0.275 | 1.516 | 1769.868 | 1.317 |
| Setsize + Probe type +  Setsize * Probe Type | 0.200 | 0.103 | 0.459 | 662.397 | 4.898 |

**Supplementary Table 4: *Bayes factor ANOVA for the Absolute Error in Experiment 2***

| Model | P(M) | P(M\|data) | BF_M_ | BF_10_ | error % |
| --- | --- | --- | --- | --- | --- |
| Null model (incl. subject) | 0.200 | 2.565e-4 | 0.001 | 1.000 |  |
| Masking | 0.200 | 0.007 | 0.027 | 26.620 | 3.997 |
| Probe Type | 0.200 | 0.007 | 0.027 | 26.610 | 1.430 |
| Masking + Probe Type | 0.200 | 0.647 | 7.332 | 2522.733 | 1.547 |
| Masking + Probe Type +  Masking * Probe Type | 0.200 | 0.339 | 2.052 | 1322.103 | 6.303 |

**Supplementary Table 5: *Bayes factor ANOVA for the Time to Initiate Responses in Experiment 2***

| Model | P(M) | P(M\|data) | BF_M_ | BF_10_ | error % |
| --- | --- | --- | --- | --- | --- |
| Null model (incl. subject) | 0.200 | 0.497 | 3.954 | 1.000 |  |
| Masking | 0.200 | 0.172 | 0.829 | 0.345 | 0.889 |
| Probe Type | 0.200 | 0.218 | 1.113 | 0.438 | 1.282 |
| Masking + Probe Type | 0.200 | 0.077 | 0.336 | 0.156 | 2.254 |
| Masking + Probe type +  Masking * Probe Type | 0.200 | 0.036 | 0.150 | 0.073 | 2.294 |

**Supplementary Table 6: *Bayes factor ANOVA for the Absolute Error in Experiment 3***

| Model | P(M) | P(M\|data) | BF_M_ | BF_10_ | error % |
| --- | --- | --- | --- | --- | --- |
| Null model (incl. subject) | 0.200 | 3.844e-12 | 1.538e-11 | 1.000 |  |
| Sequential Position | 0.200 | 0.351 | 2.166 | 9.140e+10 | 0.939 |
| Probe Type | 0.200 | 2.016e-12 | 8.063e-12 | 0.524 | 0.924 |
| Sequential Position + Probe Type | 0.200 | 0.420 | 2.900 | 1.093e+11 | 1.500 |
| Sequential Position + Probe type + Sequential Position * Probe Type | 0.200 | 0.228 | 1.184 | 5.941e+10 | 2.783 |

| *Absolute Error* | t-value | p-value |
| --- | --- | --- |
| Dot,  One – Two | 2.94 | 0.011 |
| Dot,  One – Three | 4.12 | 0.001 |
| Dot,  One – Four | 6.77 | < 0.001 |
| Dot,  Two – Three | 1.95 | 0.071 |
| Dot,  Two – Four | 4.41 | 0.001 |
| Dot,  Three– Four | 4.03 | 0.001 |
| Arrow,  One – Two | - 0.77 | 0.456 |
| Arrow,  One – Three | 0.71 | 0.489 |
| Arrow,  One – Four | 5.39 | < 0.001 |
| Arrow,  Two – Three | 1.34 | 0.201 |
| Arrow,  Two – Four | 5.33 | < 0.001 |
| Arrow,  Three– Four | 3.97 | 0.001 |

**Supplementary table 7: *Post-hoc T-Tests on absolute error by sequence position for Experiment 3***

**Supplementary Table 8: *Bayes factor ANOVA for the Time to Initiate Responses in Experiment 3***

| Model | P(M) | P(M\|data) | BF_M_ | BF_10_ | error % |
| --- | --- | --- | --- | --- | --- |
| Null model (incl. subject) | 0.200 | 0.441 | 3.160 | 1.000 |  |
| Sequential Position | 0.200 | 0.370 | 2.347 | 0.838 | 0.693 |
| Probe Type | 0.200 | 0.087 | 0.381 | 0.197 | 1.317 |
| Sequential Position + Probe Type | 0.200 | 0.072 | 0.309 | 0.162 | 1.537 |
| Sequential Position + Probe Type + Sequential Position * Probe Type | 0.200 | 0.030 | 0.124 | 0.068 | 1.271 |

**Supplementary Table 9:**

|  | Effects allowed on Target-Response and Precision | Effects allowed on Target-Response, Guessing Response and Precision | | |
| --- | --- | --- | --- | --- |
| No effect | 8274.551 | | 8260.549 |  |
| Probe effect  only | 8139.023 | | 8129.216* |  |
| Between-experiment effect  only | 8178.958 | | 8170.900 |  |
| Both effects | 8139.341 | | 8130.920 |  |
| Both effects and interaction | 8150.256* | | 8133.789 |  |
| Probe effect only,  On Precision only | 8137.275† | | 8138.295† |  |
| Probe effect only,  On Target Response only | 8160.016 | | 8169.726 |  |
| * Best model fit main analysis, † best model fit sub-analysis | | | |  |

*WAIC values of Hierarchical Bayesian measurement models for the merged dataset of the three items dot-vs-arrow conditions and the no mask, dot-vs-arrow condition of experiments 1 and 2*
